# Effect of levothyroxine treatment on fetal growth among women with mild subclinical hypothyroidism and thyroid peroxidase antibody negative: a cohort study

**DOI:** 10.1186/s12884-023-05676-5

**Published:** 2023-05-18

**Authors:** Yuelong Ji, Jinhui Xu, Tao Su, Lizi Lin, Shuang Zhou, Heling Bao, Zheng Liu, Shusheng Luo, Xiangrong Xu, Na Han, Hai-Jun Wang

**Affiliations:** 1 Department of Maternal and Child Health, School of Public Health, Peking University, Haidian District, No. 38 Xueyuan Rd, Beijing, 100191 China; 2 Tongzhou Maternal and Child Health Hospital of Beijing, Beijing, China; 3 Department of Occupational and Environmental Health, School of Public Health, Sun Yat-Sen University, Guangzhou, China; 4 School of Public Health, Peking University, Beijing, China

**Keywords:** Mild subclinical hypothyroidism, Fetal growth, Birth weight, Levothyroxine

## Abstract

**Background:**

Some clinicians used levothyroxine (LT4) treatment for mild subclinical hypothyroidism (SCH) pregnant women (2.5 < thyroid-stimulating hormone (TSH) ≤ the pregnancy-specific reference range with normal free thyroxine (FT4) level) with thyroid peroxidase antibody negative (TPOAb^−^), although the recent clinical guideline did not recommend it. It is unknown whether LT4 treatment for pregnant women with mild SCH and TPOAb^−^ have impact on fetal growth. Therefore, the aim of the study was to investigate the effect of LT4 treatment on fetal growth and birth weight among mild SCH pregnant women with TPOAb^−^.

**Methods:**

This was a birth cohort study including 14,609 pregnant women between 2016 and 2019 in Tongzhou Maternal and Child Health Hospital of Beijing, China. Pregnant women were divided into 3 groups as follows: Euthyroid (*n* = 14,285, 0.03 ≤ TSH ≤ 2.5mIU/L, normal FT4), TPOAb^−^; Untreated mild SCH with TPOAb^−^ (*n* = 248, 2.5 < TSH ≤ 2.9mIU/L, normal FT4, without LT4 treatment); Treated mild SCH with TPOAb^−^ (*n* = 76, 2.5 < TSH ≤ 2.9mIU/L, normal FT4, with LT4 treatment). The main outcome measures were Z-scores of fetal growth indicators (abdominal circumference (AC), biparietal diameter (BPD), femur length (FL), head circumference (HC), estimated fetal weight (EFW)), fetal growth restriction (FGR) and birth weight.

**Results:**

There was no difference in fetal growth indicators and birth weight between the untreated mild SCH women with TPOAb^−^ and the euthyroid pregnant women. But the HC Z-score was lower in the LT4 treated mild SCH women with TPOAb^−^, compared with the euthyroid pregnant women (β = -0.223, 95%CI: -0.422, -0.023). The LT4 treated mild SCH women with TPOAb^−^ had lower fetal HC Z-score (β = -0.236, 95%CI: -0.457, -0.015), compared with the untreated mild SCH women with TPOAb^−^.

**Conclusions:**

We observed that LT4 treatment for mild SCH with TPOAb^−^ was associated with decreased fetal HC, which was not observed for untreated mild SCH women with TPOAb^−^. The adverse effect of LT4 treatment for mild SCH with TPOAb^−^ provided new evidence for the recent clinical guideline.

**Supplementary Information:**

The online version contains supplementary material available at 10.1186/s12884-023-05676-5.

## Introduction

Maternal thyroid hormones are known to be crucial for maintaining a normal fetal growth and development, especially in the first trimester when the fetus is entirely dependent on the transplacental transport of maternal thyroid hormones [1, 2]. During the past decades, the impact of maternal thyroid diseases on maternal and fetal health has gained increasing attention. Overt thyroid dysfunctions during pregnancy are related to fetal and maternal complications, and even cause children neurodevelopment disorders in the later life [3].

Few studies have been performed to assess the association between maternal thyroid function and fetal growth in utero using ultrasound measurements [4–6]. Although birth weight was associated with infant survival [7] and children`s long-term outcomes [8], birth weight is a poor proxy for the quality of fetal growth [9]. It is a one-time measurement after birth and may not reflect fetal dynamic growth in utero over the entire pregnancy. Although the ultrasound measurement might suffer from relatively larger margin of error, the longitudinal ultrasound measurements of fetal growth indicators may truly reflect the fetal growth pattern and better identify the effect of thyroid diseases on fetal growth in a time-sensitive manner. Van Mil et al*.* [4] reported that maternal hypothyroxinemia in early pregnancy (median 13.4 week) was associated with larger fetal head size. Johns et al*.* [5] explored the association between repeatedly measured thyroid hormones and ultrasound measurements of fetal growth, observed that FT4 was inversely associated with repeated measurements of EFW, HC and AC. They didn`t observe any associations between TSH and fetal growth. However, the sample size of the study was relatively limited (*n* = 439). Whether maternal mild SCH with TPOAb^−^ have impact on fetal growth is still unclear.

Before the publication of the 2017 American Thyroid Association (ATA) guideline, the treatment of mild SCH with TPOAb^−^ were based on the 2011 ATA guideline, which did not recommend for or against LT4 treatment in mild SCH pregnant women with TPOAb^−^ due to the insufficient evidence [10]. Some clinicians used LT4 treatment for mild SCH pregnant women with TPOAb^−^, although the recent clinical guideline did not recommend it. In the recent ATA guideline in 2017, LT4 treatment is considered for mild SCH pregnant women with TPOAb positive, but LT4 treatment is not recommended for mild SCH pregnant women with TPOAb^−^ [11]. Several randomized controlled trials were carried out to investigate the effect of LT4 treatment in women with mild SCH with TPOAb^−^, none of these studies have identified beneficial effects on preventing adverse pregnancy and offspring outcomes [12–15]. Furthermore, Maraka et al. [16] reported that LT4 treatment may increase the risk of adverse pregnancy outcomes (preterm delivery, gestational hypertension and pre-eclampsia) in mild SCH pregnant women, although lacked information about TPOAb status. Zhang et al. [17] observed increased risks of gestational diabetes mellitus (GDM) in LT4 treated mild SCH women with TPOAb^−^ compared to untreated women and the controls. However, it is unknown whether LT4 treatment for pregnant women with mild SCH and TPOAb^−^ have impact on fetal growth.

Given the limited evidence, we conducted a birth cohort study to investigate the effect of LT4 treatment on fetal growth and birth weight among mild SCH pregnant women with TPOAb^−^.

## Materials and methods

### Study design and participants

The present study was based on the Peking University Retrospective Birth Cohort in Tongzhou, Beijing, which is an iodine-sufficient region in China [18]. The information of pregnant women was extracted from the electronic medical information system. Pregnant women whose last menstrual period was between May 2016 and April 2019 were included in the study.

**Fig. 1:**
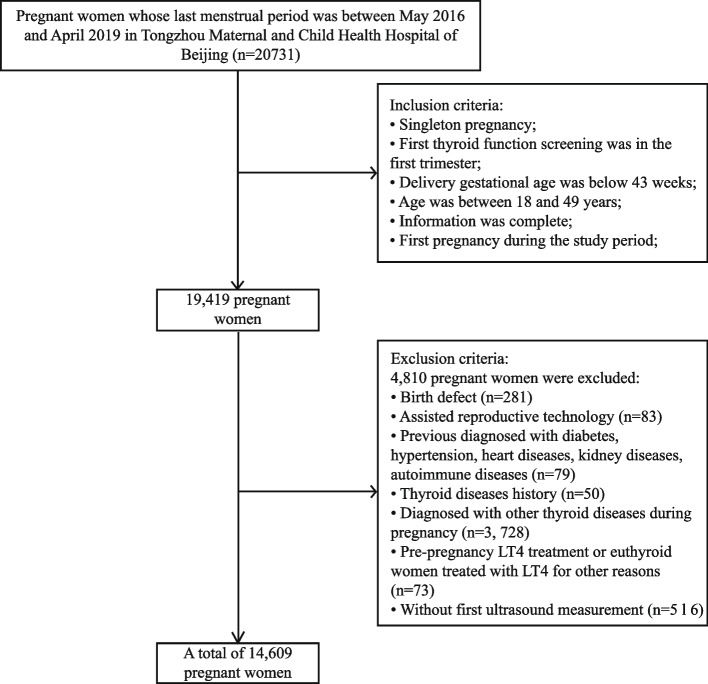
Flowchart illustrating the selection of the study population

Gestational age was estimated from the reported date of the last menstrual period. If the women had an irregular menstrual cycle, the last menstrual period was corrected by the first ultrasound measurement which was done for the confirmation of pregnancy. The inclusion criteria for subjects were: singleton pregnancy; first thyroid function screening was in the first trimester (< 14 gestational week); delivery gestational age was below 43 weeks; age was between 18 and 49 years; medical information was complete (including first prenatal visit, thyroid function screening, ultrasound examination and delivery information); for women with more than one pregnancy during the study period, the first one was selected. A total of 19,419 women were included in the birth cohort. Exclusion criteria were: the fetus had birth defects; pregnancy conceived through assisted reproductive technology; women were diagnosed with diabetes or hypertension; women had thyroid diseases history; women were diagnosed with other thyroid diseases, including hyperthyroidism, overt hypothyroidism, hypothyroxinemia, and TPOAb positive; women were treated with LT4 before pregnancy or euthyroid women were treated with LT4 during pregnancy for other reasons; women without the first ultrasound measurement (21–25 week). Finally, a total of 14,609 pregnant women were included in the present study. Figure 1 illustrated the selection of the study population. The study was approved by the Institution Review Board of Peking University Health Science Center.

### Exposure assessments and grouping

Maternal serum samples were collected when they firstly came for prenatal care in the first trimester (median gestational age = 8.9 weeks; interquartile range, IQR: 8.0–10.1 weeks), and thyroid functions were assessed using electro-chemiluminescence immunoassay with Architect i2000 (Illinois, USA), including TSH, FT4 and TPOAb. The intra-assay and inter-assay coefficients of variation (CV) of serum TSH, FT4 and TPOAb were below 10%.

According to the recommendations of ATA guideline [11] and the National Academy of Clinical Biochemistry [19], we established population-based trimester-specific reference ranges for TSH and FT4 based on the study population, in order to evaluate the thyroid function of the pregnant women. Reference ranges for maternal TSH and FT4 were defined as the range between the 2.5th and 97.5th percentiles, after exclusion of women with TPOAb positive, known thyroid disease or goiter, twin, or multifetal pregnancies. For the first trimester, the reference values were 0.03–2.9mIU/L for TSH and 10.9–18.5 pmol/L for FT4. According to the manufacturer`s instructions, TPOAb was considered positive as concentration > 5.6 IU/mL.

According to the 2017 ATA guideline [11], mild SCH was defined as 2.5mIU/L < TSH ≤ 2.9mIU/L and FT4 at the normal range (10.9 pmol/L ≤ FT4 ≤ 18.5 pmol/L), TPOAb positive was defined as TPOAb concentration > 5.6 IU/mL, euthyroid was defined as normal TSH (0.03 ≤ TSH ≤ 2.5mIU/L), FT4 (10.9 pmol/L ≤ FT4 ≤ 18.5 pmol/L), and TPOAb concentration ≤ 5.6 IU/mL.

During the study period, clinicians in the hospital diagnosed and treated thyroid diseases during pregnancy based on 2012 Chinese guidelines [20], which was based on the 2011 ATA guidelines and didn`t recommend for or against LT4 treatment among mild SCH pregnant women with TPOAb^−^. As a result, LT4 treatment decision varied from clinician to clinician, i.e., some women were treated with LT4, but others not. LT4 is a prescription drug in China and is only available in hospitals. Thus, the pregnant women received the LT4 treatment if they have LT4 prescription after thyroid function testing, which were recorded in the medical information system. The median gestational age at first LT4 treatment was 16.5 weeks (IQR: 11.5–19.4 weeks). In this study, 14,609 pregnant women were divided into 3 groups according to thyroid function and LT4 treatment status as follows: Euthyroid (*n* = 14,285): 0.03 ≤ TSH ≤ 2.5mIU/L, normal FT4; Untreated mild SCH with TPOAb^−^ (*n* = 248): 2.5 < TSH ≤ 2.9mIU/L, normal FT4, without LT4 treatment; Treated mild SCH with TPOAb^−^ (*n* = 76): 2.5 < TSH ≤ 2.9mIU/L, normal FT4, with LT4 treatment.

### Outcome measurements

Three fetal ultrasound examinations were scheduled during the routine prenatal visits at the hospital. We selected 21–25, 29–32 and 36–40 week as the interval of three ultrasound examinations, which were based on the recommendation of the Chinese guideline of prenatal care [21] and the distribution of ultrasound examinations. Among 14,609 participants, there were 10,856 (74.3%) participants with all three examinations, while 3,532 (24.2%) and 221 (1.5%) participants had two examinations and one examination during pregnancy, respectively. The fetal growth indicators (in cm) included AC, BPD, FL and HC. EFW was predicted using Hadlock`s formula: log10(EFW) = 1.326–0.00326*AC*FL + 0.0107*HC + 0.0438*AC + 0.158*FL [22]. Birth weight (in grams) were measured by trained midwife shortly after delivery.

The distribution of each fetal growth parameter depends on gestational age and gender. All fetal growth indicators were quantified as gestational age- and gender- adjusted standard deviation score (Z-score) using the Generalized Additive Models for Location, Scale and Shape (GAMLSS) [23]. The gamlss package (version 5.1.7) in R was used to calculate Z-score. First, Box-Cox transformations were used to normalize the fetal growth indicators. Then, each fetal growth parameter was modeled by a cubic spline on gestational age according to fetal gender. Finally, Z-score for all fetal growth indicators at each gestational age were calculated based on the best fitting models [24]. We defined FGR as AC or EFW Z-score < 3^rd^ centile based on clinical consensus [25]. The gestational age- and gender- adjusted birth weight Z-score were calculated by using the same method described above.

### Covariates

In the first prenatal visit, pregnant women were interviewed face-to-face by trained nurses to obtain their information, including birth date, maternal education attainment, current employment status, pre-pregnancy weight, height, parity, gynecological history, family history of diseases, last menstrual period. Maternal pre-pregnancy body mass index (BMI) was calculated as maternal pre-pregnancy weight / height^2^ (kg/m^2^). We classified pre-pregnancy BMI as underweight (BMI < 18.5 kg/m^2^), normal weight (18.5 kg/m^2^ ≤ BMI < 24.0 kg/m^2^), overweight (24.0 kg/m^2^ ≤ BMI < 28.0 kg/m^2^), and obesity (BMI ≥ 28.0 kg/m^2^) [26].

We selected a range of covariates as potential confounders based on previous literatures(4,5), including maternal age, maternal educational levels (low for high school or below, middle for vocational college, high for university or above), current employment status, parity, pre-pregnancy BMI, gestational age of thyroid function testing. Because fetal growth and birth weight Z-score already accounted for gestational age and gender, we didn`t adjusted for those variables in the analyses.

### Statistical analyses

Continuous variables were checked for normal distribution using the Kolmogorov–Smirnov test, and were presented as mean (standard deviation, SD) for normally distributed data and as median (IQR) for skewed data. Continuous variables were compared among the 3 groups by using Kruskal–Wallis *H* test, categorical variables were presented as the frequency (percentage) and compared by using the Chi-square test or Fisher exact test among the 3 groups.

Generalized estimating equation (GEE) analysis was applied to compare the Untreated and Treated mild SCH with TPOAb^−^ groups with Euthyroid group in terms of the fetal growth Z-score (using identity link function) and FGR risk (using logit link function), considering the repeated ultrasound measurements. Linear regression model was performed to compare the Untreated and Treated mild SCH with TPOAb^−^ groups with Euthyroid group in terms of birth weight Z-score. Crude model was built without adjustment for any covariates, while the adjusted model was adjusted for the covariates. To assess the effect of LT4 treatment, the Untreated and Treated mild SCH with TPOAb^−^ groups were compared, the analysis methods were similar to the preceding analyses. The model 1 was adjusted for the covariates mentioned above and the model 2 was additionally adjusted for TSH level [16].

The data analyses were performed using R software (version 4.0.2). Two-tailed *P* value < 0.05 was considered as significant.

## Results

The number and percentage of three ultrasound examinations were illustrated in Table S1. There 77.3% of mothers have three examinations. The maternal and child characteristics of the 3 groups were shown in Table 1. There were no significant differences in maternal age, maternal educational levels, pre-pregnancy BMI, gestational age at thyroid function testing, gestational age at delivery, fetal gender, and birth weight. The proportional of primipara and employment status were significant difference among Euthyroid, Untreated mild SCH and Treated mild SCH groups. The TSH level in both the Untreated and Treated mild SCH groups were significantly higher than Euthyroid group, the FT4 level in the Untreated and Treated mild SCH groups were significantly lower than Euthyroid group. There were no significant differences in TSH and FT4 levels between the Untreated and Treated mild SCH groups.

**Table 1:** Comparison of the maternal and child characteristics among the 3 groups

	Euthyroid (*n* = 14,285)	Untreated mild SCH (*n* = 248)	Treated mild SCH (*n* = 76)	*P* Value
Maternal age (years)	29.4(27.1–32.2)	29.6(27–32.8)	30(27.1–31.9)	0.833
Maternal education levels				0.116
Low (high school and below)	2200(15.4)	46(18.5)	5(6.6)	
Middle (vocational college)	6166(43.2)	109(44)	34(44.7)	
High (university and above)	5919(41.4)	93(37.5)	37(48.7)	
Current employment status				**0.045**
Yes	12,590(88.1)	219(88.3)	74(97.4)	
No	1695(11.9)	29(11.7)	2(2.6)	
Parity				**0.002**
Primipara	8029(56.2)	161(64.9) ^a^	52(68.4)	
Multipara	6256(43.8)	87(35.1) ^a^	24(31.6)	
Pre-pregnancy BMI				0.725
Underweight	1371(9.6)	21(8.5)	5(6.6)	
Normal weight	9303(65.1)	157(63.3)	47(61.8)	
Overweight	2821(19.7)	54(21.8)	18(23.7)	
Obesity	790(5.5)	16(6.5)	6(7.9)	
Gestational age at thyroid function testing (weeks)	8.9(8–10.1)	8.8(8–10.6)	8.7(7.9–10.5)	0.828
TSH (mIU/L)	0.90(0.51–1.34)	2.67(2.56–2.77) ^a^	2.71(2.61–2.81) ^b^	** < 0.001**
FT4 (pmol/L)	13.64(12.67–14.77)	12.93(12.09–13.74) ^a^	12.94(11.88–13.78) ^b^	** < 0.001**
Gestational age at delivery (weeks)	39.6(38.9–40.4)	39.9(39.1–40.7)	39.9(39–40.6)	0.058
Gender				0.073
Girls	6951(48.7)	106(42.7)	31(40.8)	
Boys	7334(51.3)	142(57.3)	45(59.2)	
Birth weight (g)	3370(3100–3660)	3365(3107.5–3680)	3395(3095–3622.5)	0.992

Continuous variables were expressed as median and IQR and compared using Kruskal–Wallis *H* test, categorical variables were presented as the frequency (percentage) and compared using the Chi-square test or Fisher exact test among the 3 groups, with post hoc Bonferroni corrections, *P* = 0.05/3

*Abbreviations*: *TSH* thyroid stimulating hormone, *FT4* free thyroxine, *BMI* body mass index

Bold indicated *P* < 0.05

^a^*P* values < 0.017 between untreated mild SCH and euthyroid

^b^*P* values < 0.017 between treated mild SCH and euthyroid

The characteristics of fetal growth indicators during pregnancy were shown in Table 2. The mean gestational age at three ultrasound measurements was 23.3, 29.9, 37.0 weeks, while the prevalence of FGR was 4.26%, 3.69%, 3.35%, respectively.

**Table 2:** Characteristics of fetal growth parameters during pregnancy

	First time (21–25 weeks, *n* = 14,609)	Second time (29-32 weeks, *n* = 13,944)	Third time (36-40 weeks, *n* = 11,300)
Gestational age at measurements (weeks)	23.3(0.68)	29.9(0.8)	37(0.72)
AC			
Original scale (cm)	19.07(0.92)	26.11(1.27)	33.05(1.54)
Z-scores	-0.03(0.98)	-0.01(0.99)	-0.02(0.98)
BPD			
Original scale (cm)	5.8(0.29)	7.74(0.33)	9.14(0.32)
Z-scores	-0.02(0.99)	0(0.98)	-0.02(0.98)
FL			
Original scale (cm)	4.13(0.19)	5.65(0.23)	6.86(0.22)
Z-scores	-0.02(0.99)	0.01(0.99)	-0.01(0.98)
HC			
Original scale (cm)	21.45(0.85)	28.07(1)	32.47(1.03)
Z-scores	-0.02(0.98)	0(0.98)	-0.02(0.98)
EFW			
Original scale(g)	614.6(67.92)	1529.21(180.68)	2935.97(304.27)
Z-scores	-0.14(0.99)	0.01(0.99)	-0.03(0.97)
FGR (frequency)	623(4.26)	514(3.69)	378(3.35)

Continuous variables were expressed as mean and standard deviation (SD)

*Abbreviations*: *AC* abdominal circumference, *BPD* biparietal diameter, *FL* femur length, *HC* head circumference, *EFW* estimate fetal weight, *FGR* fetal growth restriction

Table 3 (adjusted model) and Table S2 (crude model) showed the comparison of fetal growth and birth weight Z-scores between the Untreated or Treated mild SCH groups and Euthyroid group. After adjustment for the covariates, there was no difference in fetal HC Z-score between the untreated mild SCH women with TPOAb^−^ and the euthyroid pregnant women, but the women in Treated mild SCH with TPOAb^−^ group had lower HC Z-score than euthyroid pregnant women (β = -0.223, 95%CI: -0.422, -0.023). There was no statistically significant associations in FGR (data not shown). There were no statistically significant associations in other fetal growth indicators (AC, BPD, FL, EFW) or birth weight Z-score. In the analyses of these association by ultrasound examination period (Table S3).

**Table 3:** Comparison of fetal growth and birth weight Z-scores between the Untreated or Treated mild SCH group and Euthyroid group

	Euthyroid	Untreated mild SCH with TPOAb^−^		Treated mild SCH with TPOAb^−^	
		Adjust β (95%CI) ^a^	Adjust *P* value	Adjust β (95%CI)	Adjust *P* value
AC	ref	-0.082(-0.181, 0.017)	0.104	-0.162(-0.35, 0.027)	0.092
BPD	ref	-0.015(-0.115, 0.085)	0.773	-0.195(-0.39, 0.000)	0.050
FL	ref	-0.068(-0.164, 0.027)	0.161	-0.046(-0.241, 0.149)	0.647
HC	ref	0.012(-0.086, 0.109)	0.814	**-0.223(-0.422, -0.023)**	**0.029**
EFW	ref	-0.059(-0.157, 0.039)	0.237	-0.158(-0.36, 0.044)	0.126
Birth weight	ref	-0.048(-0.169, 0.074)	0.442	-0.06(-0.278, 0.158)	0.590

*Abbreviations*: *AC* abdominal circumference, *BPD* biparietal diameter, *FL* femur length, *HC* head circumference, *EFW* estimate fetal weight

Bold indicated *P* < 0.05

^a^The adjust model adjusted for the covariates including maternal age, maternal educational level, current employment status, parity, pre-pregnancy BMI, gestational age of thyroid function testing

We observed that the LT4 treated mild SCH women with TPOAb^−^ had lower fetal HC Z-score (β = -0.249, 95%CI: -0.471, -0.027) in the model 1. After additionally adjusted for TSH level, the association remained significant (β = -0.236, 95%CI: -0.457, -0.015). There were no association between LT4 treatment and FGR (RR = 1.77, 95%CI: 0.78, 4.04). There were no association between LT4 treatment and other fetal growth indicators (AC, BPD, FL, EFW) or birth weight Z-score (see Table 4).

**Table 4:** Comparison of fetal growth and birth weight Z-scores between the LT4 treated mild SCH and untreated mild SCH group

	Untreated mild SCH with TPOAb^−^	Treated mild SCH with TPOAb^−^			
		Model 1 ^a^		Model 2 ^b^	
		β (95%CI)	*P* value	β (95%CI)	*P* value
AC	ref	-0.120(-0.333, 0.094)	0.271	-0.129(-0.346, 0.088)	0.244
BPD	ref	-0.209(-0.424, 0.006)	0.056	-0.206(-0.422, 0.010)	0.062
FL	ref	0.010(-0.205, 0.226)	0.925	-0.007(-0.226, 0.212)	0.951
HC	ref	**-0.249(-0.471, -0.027)**	**0.028**	**-0.236(-0.457, -0.015)**	**0.036**
EFW	ref	-0.136(-0.360, 0.087)	0.233	-0.146(-0.373, 0.080)	0.206
Birth weight	ref	-0.057(-0.317, 0.204)	0.670	-0.065(-0.329, 0.199)	0.628

*Abbreviations*: *AC* abdominal circumference, *BPD* biparietal diameter, *FL* femur length, *HC* head circumference, *EFW* estimate fetal weight

Bold indicated *P* < 0.05

^a^The model 1 was adjusted for the covariates including maternal age, maternal educational levels, current employment status, parity, pre-pregnancy BMI and gestational age of thyroid function testing

^b^The model 2 was additionally adjusted for TSH level

## Discussion

The present study investigated the effect of LT4 treatment on fetal growth and birth weight among mild SCH pregnant women with TPOAb^−^. We found that there was no difference in fetal growth indicators and birth weight between the untreated mild SCH women with TPOAb^−^ and the euthyroid pregnant women, but the LT4 treated mild SCH women with TPOAb^−^ had lower fetal HC Z-score than the untreated mild SCH women with TPOAb^−^ and euthyroid women.

In this study, we did not find that untreated mild SCH were associated with birth weight, which were consistent with previous studies [27–29]. Carty et al*.* [27] found that there were no differences in fetal birth weight between pregnant women with TSH 2.5-5mIU/L and those with TSH < 2.5mIU/L. Hadar et al. [28] conducted a retrospective cohort study and reported no association between TSH 2.5–4.0mIU/L and birth weight in the first trimester. Li et al. [29] also found that a mildly elevated TSH level (2.5–4.0mIU/L) during the first trimester in TPOAb^−^ pregnant women was not associated with birth weight.

Birth weight is a crude measure of fetal intrauterine growth at the endpoint of pregnancy, and it cannot provide information for the possible growth impairment at specific intrauterine period. The growth impairment in utero could induce long-term complications [30, 31]. Longitudinal ultrasound measurements of fetal growth indicators can prospectively reflect the intrauterine fetal growth and better capture the effect of thyroid diseases on fetal growth in a time-sensitive manner. To our best knowledge, this was the first study to investigate the association between maternal mild SCH with or without LT4 treatment and fetal growth in utero. Although we were unable to compare our results with other studies directly, there were some studies investigating the association of maternal TSH with fetal growth. Johns et al. [5] explored the association between repeatedly measured thyroid hormone and ultrasound measurements, and didn`t observed any associations between TSH and repeated measurements of EFW, HC and AC. Another study in China involving 46,186 mothers indicated that there was significantly negative association between TSH and CRL in the first trimester [6]. Our findings together with previous literature revealed the association between maternal TSH and fetal growth. Further studies are needed to elucidate the underlining biological pathways.

In the present study, we found that LT4 treatment for mild SCH with TPOAb^-^ was associated with decreased fetal HC Z-score. Although the transfer process of additional LT4 dosage from mothers to fetal blood through the utero-placental unit is still unclear [32], a previous study showed that FT4 concentrations were higher than normal levels in around 60% of fetuses whose mothers were euthyroid with autoimmune thyroid disease and received LT4 treatment [33]. Results from previous studies consistently indicated that the high FT4 levels in maternal blood were associated with decreased fetal growth indicators [5, 6]. Thyroid hormones potentially have a U-shape effect on fetal brain development, both the lack or excess of thyroid hormone during pregnancy might impair fetal brain development [34]. A population-based prospective cohort study investigated the association of maternal thyroid hormone with child brain morphology and found that high maternal FT4 concentrations have been associated to lower grey matter and cortex volume [35]. Our study results indicate that the LT4 treatment for mild SCH with TPOAb^-^ had lower fetal HC, which may be due to high FT4 levels in mothers and affect fetal brain development. According to 2017 ATA guideline, LT4 treatment is not recommended for mild SCH with TPOAb^-^ with high-quality evidence. But the guideline had no evidence for adverse effect of LT4 treatment on fetal growth. Our findings for the adverse effect of LT4 treatment provided new evidence to support the recent ATA guideline for mild SCH with TPOAb^-^ [11].

The strength of this study included the large sample and the availability of longitudinal ultrasound measurements of fetal growth, which allowed us to investigate the association between mild SCH with or without LT4 treatment and fetal growth for the first time. In addition, our study hospital used 2012 Chinese guidelines [20] for the diagnosis and treatment of maternal thyroid diseases during our study period. Since the 2012 Chinese guidelines did not recommend for or against LT4 treatment, not every mild SCH women with TPOAb^−^ were treated. This setting provided us the opportunity to compare the LT4 treated and untreated groups to evaluate the adverse effect of LT4 treatment among those women.

The limitations of this study were the following. First, this is a longitudinal observational study instead of randomized clinical trial. Whether mild SCH women with TPOAb^−^ were treated or untreated with LT4 treatment was dependent on the clinical practice of different clinicians. We compared the maternal characteristics between the treated and untreated group, however, did not find significant differences. Still, the difference in numbers of treated and untreated patients might affect the robustness of our findings. Thus, the observational nature of this study limits causal inference. Secondly, the LT4 treatment was defined by prescription of LT4 in the medical information system, we lacked data for LT4 adherence and continuous follow-up of thyroid function. Thirdly, due to the limited sample size for the mild SCH, our findings were only marginally significant after adjusting for multiple testing. Larger studies are needed to further confirm our findings. Finally, neonatal head circumference data is not available for current study. Future study could use neonatal head circumference to further test our findings.

## Conclusions

In conclusion, we observed that LT4 treatment for mild SCH with TPOAb^−^ was associated with decreased fetal HC Z-score, which was not observed for untreated mild SCH women with TPOAb^−^. The potential adverse effect of LT4 treatment for mild SCH with TPOAb^−^ provided new evidence to support the recent clinical guideline. Further studies are needed to elucidate the biological mechanisms underlying the effect LT4 treatment for maternal mild SCH on fetal growth.

## Supplementary Information

**Additional file 1: Supplemental Tables. Table S1.** Number and percentage of three ultrasound examinations. **Table S2. **Association of untreated and treated mild SCH with fetal growth and birth weight Z-scores (crude model). **Table S3.** Association of untreated and treated mild SCH with fetal growth and birth weight Z-scores by examination period.

## Data Availability

The datasets generated and/or analyzed during the current study are not publicly available due to the Biosecurity Law of the P.R.C. but are available from the corresponding author on reasonable request.

## Abbreviation

LT4: Levothyroxine
