# Supplementary material for: Effect of levothyroxine treatment on fetal growth among women with mild subclinical hypothyroidism and thyroid peroxidase antibody negative: a cohort study
Source: BMC Pregnancy Childbirth. 2023 May 18;23:362. doi: 10.1186/s12884-023-05676-5 (PMC10193713; doi:10.1186/s12884-023-05676-5)
Supplement: Supplementary file 1 — Additional file 1: Supplemental Tables. Table S1. Number and percentage of three ultrasound examinations. Table S2. Association of untreated and treated mild SCH with fetal growth and birth weight Z-scores (crude model). Table S3. Association of untreated and treated mild SCH with fetal growth and birth weight Z-scores by examination period. [file 12884_2023_5676_MOESM1_ESM.docx]

**Additional file 1: Supplemental Materials**

**Supplemental Tables:**

Table S1 Number and percentage of three ultrasound examinations

Table S2 Association of untreated and treated mild SCH with fetal growth and birth weight Z-scores (crude model).

Table S3 Association of untreated and treated mild SCH with fetal growth and birth weight Z-scores by examination period.

**Table S1** Number and percentage of three ultrasound examinations

|  | 21-25 week | 29-32 week | 36-40 week |
| --- | --- | --- | --- |
| All | 14609 | 13944 (95.4) | 11300 (77.3) |
| Euthyroid | 14285 | 13631 (95.4) | 11058 (77.4) |
| Untreated mild SCH | 248 | 239 (96.3) | 195 (78.6) |
| Treated mild SCH | 76 | 74 (97.3) | 47 (61.8) |

**Table S2** Association of untreated and treated mild SCH with fetal growth and birth weight Z-scores (crude model).

|  | Euthyroid | Untreated mild SCH | | Treated mild SCH | |
| --- | --- | --- | --- | --- | --- |
|  |  | Crude β  (95%CI) ^a^ | Crude P value | Crude β  (95%CI) | Crude P value |
| AC | ref | -0.075(-0.175, 0.024) | 0.138 | -0.153(-0.345, 0.039) | 0.119 |
| BPD | ref | -0.004(-0.106, 0.098) | 0.936 | -0.171(-0.364, 0.023) | 0.084 |
| FL | ref | -0.07(-0.167, 0.027) | 0.159 | -0.045(-0.243, 0.154) | 0.659 |
| HC | ref | 0.018(-0.081, 0.116) | 0.726 | -0.208(-0.41, -0.007) | 0.043 |
| EFW | ref | -0.054(-0.153, 0.045) | 0.288 | -0.148(-0.355, 0.058) | 0.158 |
| Birth weight | ref | -0.048(-0.171, 0.076) | 0.452 | -0.049(-0.272, 0.173) | 0.663 |

Abbreviations: AC, abdominal circumference; BPD, biparietal diameter; FL, femur length; HC, head circumference; EFW, estimate fetal weight.

^a^ The crude model without adjustment for any covariate.

Table S3 Association of untreated and treated mild SCH with fetal growth and birth weight Z-scores by examination period.

|  | 21-25 week | | 29-32 week | | 36-40 week | |
| --- | --- | --- | --- | --- | --- | --- |
|  | Adjust β or OR (95%CI) ^a^ | Adjust P value | Adjust β or OR (95%CI) ^a^ | Adjust P value | Adjust β or OR (95%CI) ^a^ | Adjust P value |
| EFW | -0.07(-0.334,0.195) | 0.605 | -0.236(-0.503,0.031) | 0.084 | -0.197(-0.488,0.094) | 0.186 |
| AC | -0.044(-0.318,0.229) | 0.751 | -0.195(-0.461,0.071) | 0.152 | -0.191(-0.489,0.107) | 0.210 |
| BPD | -0.077(-0.322,0.168) | 0.538 | -0.334(-0.612,-0.055) | 0.019 | -0.339(-0.656,-0.022) | 0.037 |
| FL | -0.009(-0.273,0.254) | 0.944 | -0.077(-0.335,0.182) | 0.562 | 0.018(-0.282,0.318) | 0.905 |
| HC | -0.146(-0.396,0.104) | 0.254 | -0.391(-0.665,-0.118) | 0.005 | -0.276(-0.582,0.029) | 0.077 |
| FGR | 1.88(0.56,6.37) | 0.310 | 1.99(0.64,6.24) | 0.237 | 1.28(0.07,22.53) | 0.868 |

Abbreviations: AC, abdominal circumference; BPD, biparietal diameter; FL, femur length; HC, head circumference; EFW, estimate fetal weight.

^a^ The adjust model adjusted for the covariates including maternal age, maternal educational level, current employment status, parity, pre-pregnancy BMI, gestational age of thyroid function testing.
